# Evidence-based medicine

**DOI:** 10.1590/1516-3180.2018.136260318

**Published:** 2018-01-09

**Authors:** Álvaro Nagib Atallah

**Affiliations:** I Full Professor and Head of the Discipline of Emergency Medicine and Evidence-Based Medicine, Universidade Federal de São Paulo (UNIFESP), and Director of Cochrane Brazil, São Paulo (SP), Brazil.

Evidence-based medicine (EBM) originated from the new science that resulted from combining the methods of epidemiology and clinical research. At a time when clinicians and epidemiologists were beset by rivalry, Archibald Cochrane integrated knowledge from these two fields and created what came to be known as clinical epidemiology. With help from his collaborators, including Professor Kerr L. White, he set out his clamor for efficacy, effectiveness and efficiency in clinical teaching, practice and research.

EBM crowns the fundamental concepts of medicine and healthcare through requiring evidence of effectiveness, efficiency and safety to guide decision-making such that there will be greater likelihood of making correct decisions. The concept of EBM was introduced at the beginning of the 1990s and was followed by establishment of the Cochrane Collaboration in 1992,by Professor Iain Chalmers in Oxford.^1^

Cochrane Brazil was founded in 1996 (under the name “Centro Cochrane do Brasil”, i.e. Cochrane Center of Brazil), one year after I was first elected as Scientific Director of the São Paulo Medical Association (Associação Paulista de Medicina, APM). In the same year, the postgraduate course that today is named the Postgraduate Program on Evidence-Based Healthcare was founded at the São Paulo Medical School (Escola Paulista de Medicina), Federal University of São Paulo (Universidade Federal de São Paulo, UNIFESP).

Supporting medical practices on the basis of the best evidence, coming from rigorous methodologies that give this evidence comparative validity, is a civilizing process. This process was started by René Descartes at the beginning of the 18^th^ century and was followed by publication of the first controlled clinical trial by James Lind.^2^ Lind’s work brought in the cure for scurvy and changed the course of human history. Moreover, it changed Brazilian history through avoidance of thousands of deaths among professionals of strategic importance for this country’s development, who were brought to Brazil by Dom João VI in 1808.^3^ In 1834, Alexander Louis went against the prevailing practice of treating all diseases through bloodletting, by conducting comparative studies (with or without bloodletting). He found that there was no benefit from this cruel practice, which was based on fanciful beliefs and on the authority of certain individuals who called themselves doctors.^4^^,^^5^

In 1948, Bradford Hill and Archibald Cochrane conducted the first blinded randomized controlled clinical trial, to test use of streptomycin for treating tuberculosis.^6^ The study design in itself represented a revolution within medicine, since it created randomization and started “blinded” reading of radiographs by three observers. Furthermore, this study provided the first description of the cure for tuberculosis. In this manner, blinded randomized clinical trials became the research design model for comparing any proposed new treatment with placebo initially, if ethical, and subsequently with the traditional treatment when necessary.

The entire new methodology that was proposed had the objective of rationalizing how studies were conducted, with the aim of supplanting the bias caused through fantasies, emotions, beliefs and interests. In particular, it aimed to combat financial interests: not only among individuals but also among major corporations that seek to make profits. Few attitudes can lead to so much efficiency and avoid so much suffering and waste of money and lives as the use of evidence for making decisions, not only in relation to human health and the right to healthcare but also in relation to education, agriculture, veterinary science, social science and so on.

Deviation from this civilizing path is unthinkable. To recommend treatments without any scientific basis would be equivalent to regressing to the year 1650. It would throw back medical care by centuries, waste money, opportunities and lives and represent choosing irresponsible management of all these values.

In the postgraduate program at UNIFESP, more than ­300 ­master’s and doctoral students have now graduated, to become involved in teaching and research. The Cochrane Library has now published more than 8,000 systematic reviews that map out important subjects relating to prevention, diagnosis and treatment of greater efficiency and safety for human health. Moreover, it has published the references of almost one million randomized clinical trials, which it has made available to everyone.

With support from the APM and from the Coordination Office for Improvement of Higher-Education Personnel (Coordenação de Aperfeiçoamento de Pessoal de Nível Superior, CAPES),Cochrane Brazil has been placing all this information at the disposal of all Brazilians since 2001. This information is available simply by accessing the website of Cochrane Brazil (www.cochranebrazil.org.br).

We will be returning to this topic with further details.
